# Ground penetrating radar data used in discovery of the early Christian church of Notre Dame de Baudes near Labastide-du-Temple, France

**DOI:** 10.1016/j.dib.2016.04.057

**Published:** 2016-04-30

**Authors:** Ted L Gragson, Victor D. Thompson, David S. Leigh, Florent Hautefeuille

**Affiliations:** a Department of Anthropology, University of Georgia, Athens, USA; b TRACES (UMR 5608), Université Toulouse - Jean Jaurès, France; c Department of Geography, University of Georgia, Athens, USA

**Keywords:** Southwest France, Tarn River, Ground-penetrating radar, Medieval archeology, Site discovery

## Abstract

Data on ground-penetrating radar transect files are provided that support the research presented in "Discovery and Appraisal of the Early Christian Church of Notre Dame de Baudes near Labastide-du-Temple, France" [1]. Data consist of 102 transect files obtained with a GSSI SIR-3000 controller and a 400 MHz center frequency antenna in two grid blocks covering ca. 2700 m^2^. The data are distributed raw without post-processing in SEG-Y rev. 1 format (little endian).

**Specifications Table**

**Table:** 

Subject area	*Archaeology*
More specific subject area	*Medieval Archaeology of Southwest France*
Type of data	*Figure, table*
How data was acquired	*GSSI SIR-3000 controller with a 400 MHz center frequency antenna with encoder survey wheel*
Data format	*Raw*
Experimental factors	*The data are provided as collected without post-treatment*
Experimental features	*Data were collected in two grid blocks using parallel lines spaced 0.5 m apart with 512 samples per trace and an 80 ns time range*
Data source location	*1000 m NNW (44° 5′ N, 1° 11′ E) of the town of Labastide-du-Temple (82100), Tarn-et-Garonne department, France*
Data accessibility	*Data are with this article*

**Value of the data**

• Data can be used to identify the position, orientation and approximate dimensions of a medieval rural Christian church established no later than AD 1100.
• Data cover an area of ca. 2700 m^2^ and can be used to identify sedimentary stratigraphy of this portion of the floodplain of the Tarn River.
• The data are interesting as much for their archeological as for their geomorphic content as the area has been subject to regular flooding throughout the Holocene and the burial process of the church by alluvium is evident in the data.
• The data can be used for advanced undergraduate and graduate student training in how to prepare and analyze ground-penetrating radar data obtained in fine-textured silty alluvial sediments.

## 1 Data

With this article we provide 102 ground-penetrating radar transect files covering ca. 2700 m^2^ obtained with a 400 MHz center frequency antenna. These are raw data files in SEG-Y rev. 1 Data Exchange Format (little endian) collected during the first year of a multi-year systematic archeological siteless survey of the interfluve between the Garonne and Tarn rivers [2]. In-depth analysis of the data is presented in the associated research article [1] along with historical and sediment auger results.

## 2 Experimental design, materials and methods

**Fig. 1:**
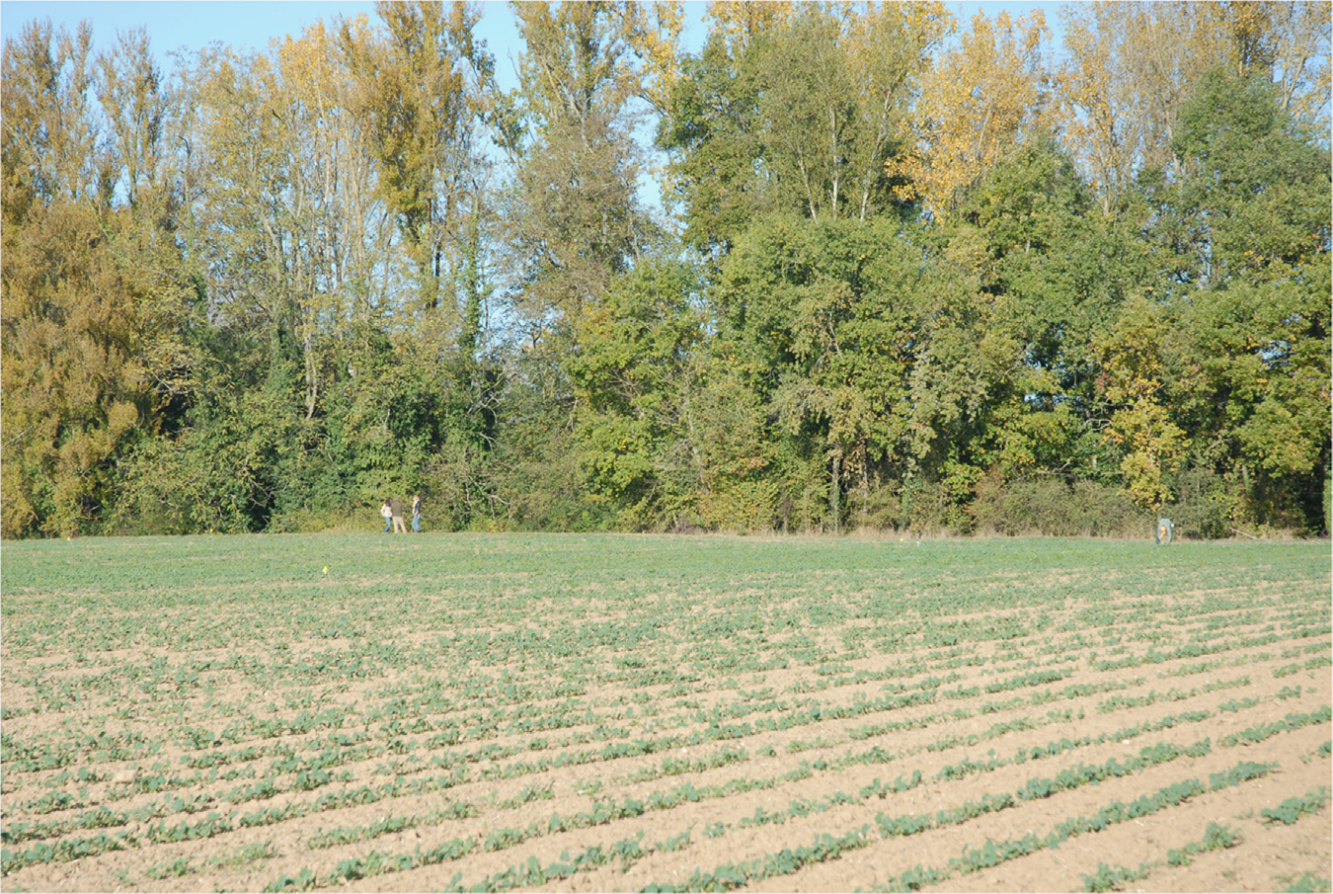
Looking NW across the site as Grid Block 1 was being laid out. The location with a slightly higher count of brick fragments and a slight elevation above the grade of the field lies in the mid-foreground in line with the group of three individuals in the background by the forest edge.

Data were collected October 30–31, 2014. These were clear-sky days with a mean average temperature of 16 °C (range: 8–24 °C) and a mean dew point of 50 °C (range: 8–13 °C). It had not rained during the previous 10 days and the fine-textured silty alluvial sediments were moist, but not wet. The field where the survey was conducted had been recently plowed, harrowed and planted in rape that was just sprouting (<2 cm tall). We first conducted a pedestrian survey of the field in search of a surface debris pattern, and discovered two locations (ca. 5 m diameter) with a slight tint suggesting the soil might contain ash or charcoal powder. The area within the two locations (ca. 30 m diameter) had a brick fragment count (ubiquitous throughout the region) of 1 per 25 m^2^ versus the surrounding area where it was ca. 1 per 100 m^2^. From a distance, the area appeared to be slightly elevated (<20 cm) relative to the nearly flat field (Fig. 1). We used this evidence along with documentary information that a church existed somewhere in the zone to carry out the ground-penetrating radar survey and auger testing.

We used a GSSI SIR-3000 control unit with a 400 MHz center frequency antenna connected to an encoder survey wheel. Two grid blocks were set up with parallel lines spaced 0.5 m apart in which 512 samples were recorded per trace using an 80 ns time range. Blocks and lines were positioned using measuring tapes, surveyor ropes and flagging. The four corners of each block were recorded with a Trimble 3000 series GeoXH at a nominal horizontal accuracy of <1 m. Profiles were collected in sinuous (zigzag) pattern moving the antenna in opposite directions on each parallel transect. Grid Block 1 was 40×60 m and centered on the mounded area with the tinted soil and slightly elevated brick count. Grid Block 2 was 10×35 m, adjacent and to the north of Grid Block 1, but with survey lines perpendicular to those in Grid Block 1.

**Fig. 2:**
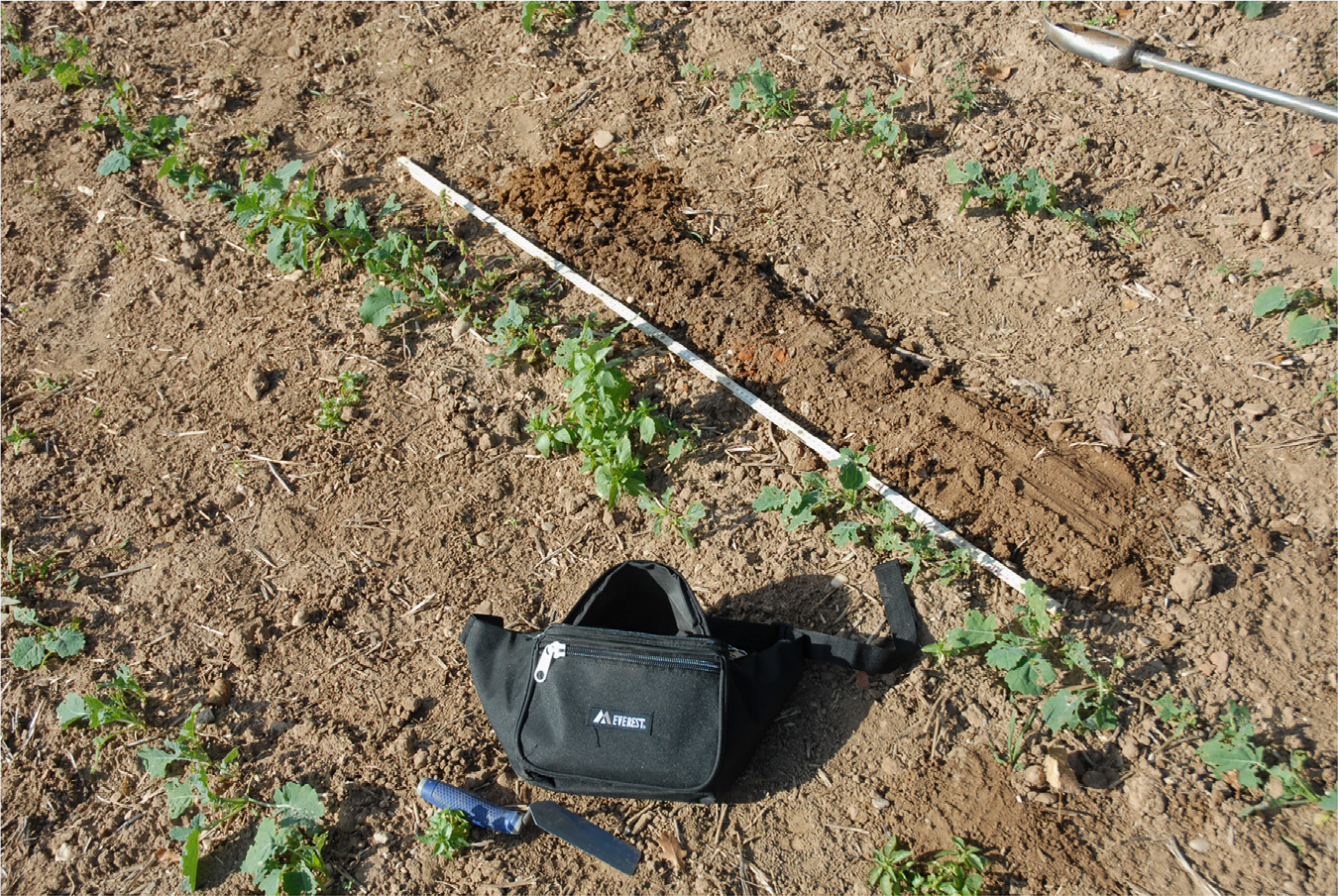
Close up taken in Grid Block 1 showing size of rape plants, crop row separation of ca. 50 cm, surface condition, and a 90 cm auger sediment sample.

Survey lines were oriented in each grid to follow the crop rows and avoid damage to the rape sprouts (Fig. 2). This meant we were not able to perfectly edge-match the two grids, and the presence of crop rows leads to some coupling errors in the data. Grid Block 1 lies completely within the agricultural field. Grid Block 2, however, extends into the farm road (hard packed earth) that bounds the north side of the field and the response difference between the field and the road is evident in several of the initial transect files for Grid Block 2.

The church remains were discovered in a cumulic buried A horizon between 40–110 cm deep. The dielectric constant estimated in the field was 8.00 (velocity=10.60 cm/ns). However, using midpoint measurements on diffraction hyperbolae across multiple radargrams combined with measured depths in multiple auger holes we determined the dielectric constant was 12.17 (velocity=8.59 cm/ns). The workflow steps that we followed in the analysis of the data are described in Gragson et al. [1].

The original files are in proprietary RADAN format, but we make the individual transect files available in SEG-Y ver. 1 [3]. This is an open standard format developed by the Society of Exploration Geophysicists in 1975 [4], and subject to various legacy constraints as a file format from the days of IBM mainframes and punchcards. Nevertheless, SEG-Y rev. 1 remains the preferred preservation file format for GPR data [5]. The files have not been post-processed other than to translate them to SEG-Y rev. 1 format. To translate the files we used Reflex2D Quick 2.5 [6] with the following settings: export format SEGY-DOS, scaling factor for coordinates=1, and output parameters *segy_ibm_format* and *ps timeincr* checked. This SEG-Y variant produces a file with unswapped (little endian) byte order and IBM 32-bit floating-point numbers.

File structure was validated using SeiSee (ver. 2.22.5), a freeware seismic data-viewing program developed by Sergey Pavlukhin [7]. We imported the files back into Reflex-W as well as into GPR-Viewer and GPR-Slice to validate the structure before distributing them. The RADAN header information does not import into the SEG-Y header, but the parameters are useful for initial processing of the files and we provide this information in the format of a SEG-Y ASCII header (Supplementary Table).

### 2.1 Coordinate space

**Fig. 3:**
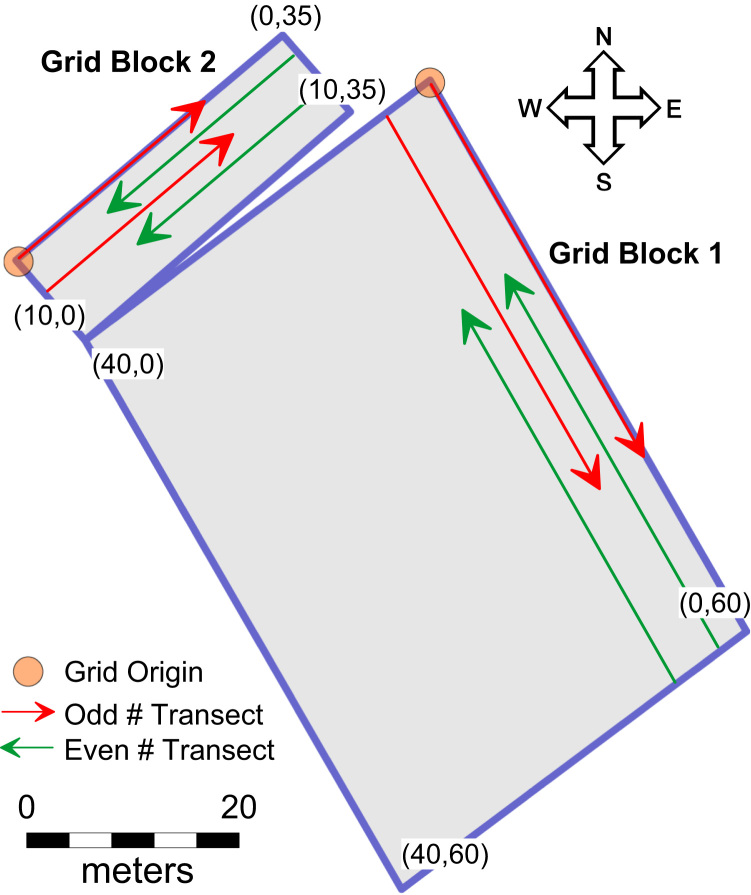
Grid block coordinate space and geography showing transect line orientations.

Grid Block 1 (GB1): *X*=0−40 m, *Y*=0−60 m. Origin=(0,0) is located in the lower left corner of the grid, which is NE geographically. In collecting transect data, the antenna was moved along the *Y*-axis of the grid. Odd numbered lines (and files) where collected travelling from *Y*=0 to *Y*=60 on a constant *X* (SE), while even numbered lines were collected traveling from *Y*=60 to *Y*=0 on a constant *X* (NW, Fig. 3). There are a total of 81 transect files in this grid block, and their numbers increase from E to W beginning with GB1_Scan01 (Supplementary Directory). Geographic coordinates of the corners of both grid blocks are projected into UTM Zone 31 N, WGS84 (Table 1).

Grid Block 2 (GB2): *X*=0−10 m, *Y*=0−35 m. Origin=(0,0) is located in the lower left corner of the grid, which is NW geographically. In collecting transect data, the antenna was moved along the *Y*-axis of the grid. Odd numbered lines (and files) were collected traveling from *Y*=0 to *Y*=35 on a constant *X* (NE), while even numbered lines where collected traveling from *Y*=35 to *Y*=0 on a constant *X* (SW). There are a total of 21 transect files in this grid block, and their numbers increase from N to S beginning with GB2_Scan01 (Supplementary Directory). The long-axis of Grid Block 2 (*Y*-axis, oriented NE-SW) is at right angles to the long-axis (*Y*-axis, oriented NW-SE) of Grid Block 1, while scan line 21 of Grid Block 2 parallels the *X*-axis of Grid Block 1.

## Figures and Tables

**Table 1:** Grid to geography coordinate space correspondence, UTM Zone 31 N, WGS84, meters.

**Grid Block 1 (GB1)**			**Grid Block 2 (GB2)**		
***(X,Y)***	***E***	***N***	***(X,Y)***	***E***	***N***
(0,0)	355309	4883849	(0,0)	355270	4883832
(0,60)	355339	4883797	(0,35)	355295	4883853
(40,60)	355307	4883772	(10,35)	355301	4883846
(40,0)	355277	4883824	(10,0)	355277	4883824
