# Supplementary material for: Ground penetrating radar data used in discovery of the early Christian church of Notre Dame de Baudes near Labastide-du-Temple, France
Source: Data Brief. 2016 Apr 30;7:1588–93. doi: 10.1016/j.dib.2016.04.057 (PMC4866399; doi:10.1016/j.dib.2016.04.057)
Supplement: Supplementary file 2 — Supplementary material [file mmc2.docx]

Table 1. ASCII reel identification header for transect line files.

| C 1 | ND Baudes Site, 1000 m NNW of Labastide-du-Temple, FR (44D5M N, 1D11M E) |
| --- | --- |
| C 2 | File Creation Date: Oct 30-31, 2014 |
| C 3 | File Modification Date: February 28, 2016 |
| C 4 |  |
| C 5 |  |
| C 6 | Horizontal Parameters |
| C 7 | Scans/Sec: 120.00 |
| C 8 | Scans/Unit (m): 50.000 |
| C 9 | Units/Mark (m): 2.000 |
| C10 |  |
| C11 | Vertical Parameters |
| C12 | Samples/Scan: 512 |
| C13 | Bits/Sample: 16 |
| C14 | Dielectric Constant - field: 8.00 (vel. 10.60 cm/ns) |
| C15 | Dielectric Constant - measured: 12.17 (vel. 8.59 cm/ns) |
| C16 |  |
| C17 | Collection Parameters |
| C18 | Control Unit: GSSI SIR-3000 |
| C19 | Antenna Type: 400 MHz center frequency |
| C20 | Distance: encoder survey wheel |
| C21 | Position (ns): 0.00 |
| C22 | Range (ns): 50.00 |
| C23 | Top Surface (m): -0.265 |
| C24 | Depth (m): 2.652 |
| C25 |  |
| C26 | Range Gain |
| C27 | # of Points: 3 |
| C28 | Gain 1: -20.00 |
| C29 | Gain 2: 50:00 |
| C30 | Gain 3: 64.00 |
| C31 |  |
| C32 | Position Correction |
| C33 | Shift (ns): 7.60 |
| C34 |  |
| C35 | IIR Filters |
| C36 | Vertical (MHz) |
| C37 | Low Pass: 800 |
| C38 | High Pass: 100 |
| C39 |  |
| C40 | E<D EBCDIC |
